# Mucoid degeneration of the anterior cruciate ligament: a scoping review

**DOI:** 10.1186/s12891-026-10327-9

**Published:** 2026-08-11

**Authors:** Thomas Vingerhoets, Wouter Platteau, Kwinten Vingerhoets, Imrane Gazali

**Affiliations:** 1 Department of Orthopaedic Surgery, Antwerp University Hospital (UZA), Edegem, Belgium; 2 Faculty of Medicine and Health Sciences, University of Antwerp, Antwerp, Belgium; 3 Faculty of Medicine, KU Leuven, Louvain, Belgium

**Keywords:** Anterior cruciate ligament, Arthroscopy, Ganglion cysts, Knee pain, Magnetic resonance imaging, Osteoarthritis, Knee

## Abstract

**Background:**

Mucoid degeneration of the anterior cruciate ligament (MD-ACL) is an uncommon but increasingly recognised cause of atraumatic knee pain and terminal motion loss. Three earlier systematic reviews separately addressed management outcomes, debridement technique, and morphometric associations, but none integrated these domains with recent histopathological, transcriptomic, and longitudinal imaging evidence, while newer conservative-management reports have added limited preliminary clinical data. This scoping review aimed to map and synthesise the available evidence on MD-ACL across terminology, epidemiology, pathophysiology, histopathology, clinical presentation, imaging, differential diagnosis, arthroscopic findings, treatment, and outcomes.

**Methods:**

PubMed/MEDLINE and Embase were searched from database inception to 31 January 2026, following the Preferred Reporting Items for Systematic Reviews and Meta-Analyses extension for Scoping Reviews (PRISMA-ScR) and the framework of Arksey and O’Malley as refined by Levac et al. Studies reporting clinical, radiological, histopathological, operative, or outcome data on MD-ACL in human subjects were eligible. Two reviewers screened titles, abstracts, and full texts independently and in duplicate; disagreements were resolved by consensus involving a third reviewer. Findings were synthesised narratively.

**Results:**

Fifty-four publications were included, comprising case reports (*n* = 7), case series (*n* = 12), retrospective cohort or comparative studies (*n* = 20), prospective cohort studies (*n* = 3), one controlled clinical trial, three systematic reviews, five technical notes, and three narrative reviews. MRI typically demonstrated a thickened, hyperintense but continuous ligament, often with the celery stalk sign. Increased posterior tibial slope was the most consistently replicated morphometric association, whereas findings on notch width index were less uniform. Concomitant meniscal and chondral pathology was common. Arthroscopic partial debridement, with or without notchplasty, generally improved pain and range of motion, but postoperative laxity became more likely with more extensive resection.

**Conclusions:**

MD-ACL is a recognised cause of atraumatic posterior knee pain in middle-aged adults with an otherwise stable knee. Current data support an association with meniscal and cartilage pathology, but do not establish a causal link with osteoarthritis progression. Prospective comparative studies with standardised diagnostic definitions and outcome reporting are needed to guide treatment more definitively.

**Supplementary Information:**

The online version contains supplementary material available at https://doi.org/10.1186/s12891-026-10327-9.

## Background

Diffuse intraligamentous signal change within an intact anterior cruciate ligament (ACL) was first linked to mucoid and eosinophilic degeneration on MRI-histological correlation in cadaveric specimens by Hodler et al. in 1992 [1]. The clinical syndrome itself was subsequently characterised by Kumar et al. in 1999 [2], who described an intact but mucinously infiltrated ligament causing posterior knee pain and restricted terminal range of motion in the absence of instability. With wider use of magnetic resonance imaging (MRI), it has become clear that diffuse signal abnormality within an intact, normally oriented ACL does not necessarily indicate rupture. A characteristic MRI appearance, often termed the celery stalk sign, reflects preserved low-signal fibres traversing diffuse intraligamentous mucoid change [3, 4].

The condition nevertheless remains under-recognised in routine practice because its MRI appearance may overlap with partial ACL tearing and because symptoms often coexist with meniscal and osteoarthritic pathology [3, 5, 6]. This overlap creates two distinct forms of misclassification: an intact but signal-abnormal ligament may be mistaken for a partial tear and referred for unnecessary reconstruction, or mucoid degeneration of the ACL (MD-ACL) may go unrecognised as a distinct contributor to symptoms, delaying targeted arthroscopic treatment. Three systematic reviews have addressed MD-ACL to date, each focused on a single domain: Sweed et al. summarised postoperative pain relief and instability risk across surgical series [7], Vivekanantha et al. characterised the range of reported debridement techniques [8], and in a separate review the same group identified posterior tibial slope, notch width index, and tibial tuberosity-trochlear groove distance as morphometric factors associated with MD-ACL [9]. All three of these reviews are charted individually among this scoping review's 54 included publications; their underlying primary studies were charted separately from the original publications, and pooled review-level outcome data were not combined with individually charted primary-study data, to avoid double-counting. None of these reviews integrated their respective domains with one another or with the further developments that have emerged since: histopathological and transcriptomic characterisation of the ligament itself, which reframes MD-ACL as an active remodelling process rather than passive degeneration [10]; longitudinal Osteoarthritis Initiative (OAI) analyses linking MD-ACL to structural disease progression [11, 12]; and newer conservative-management and longer-term outcome series that have added clinical data, although evidence for non-operative treatment remains limited [13, 14]. No existing review has integrated these mechanistic, epidemiological, morphometric, and clinical strands, and how they relate to one another remains unresolved, including whether MD-ACL is best understood as a primarily biomechanical phenomenon, a marker of a broader degenerative process, or both.

A scoping review, rather than a further systematic review, is the appropriate design for this synthesis. The literature has expanded into evidence types that are not amenable to pooled treatment-effect synthesis, including case reports, small surgical series, imaging studies, histopathological and transcriptomic analyses, technical notes, comparative cohorts, and secondary reviews; a review restricted to a single comparative question, such as treatment outcome, cannot map how these evidence types relate to or constrain one another. This scoping review therefore addressed the following research question: what is the available evidence on MD-ACL with respect to (Population) adolescent and adult patients with confirmed or suspected MD-ACL, (Concept) the domains of definition and terminology, epidemiology, pathophysiology, histopathology, clinical presentation, imaging, differential diagnosis, arthroscopic findings, management strategies, and clinical outcomes, and (Context) all clinical settings, including outpatient, arthroscopic, and arthroplasty contexts?

Accordingly, this review aimed not simply to catalogue what has been published since the prior systematic reviews, but to integrate clinical, imaging, morphometric, histopathological, and molecular evidence into one coherent picture, to identify where this evidence converges and where genuine controversy remains, and to map and narratively synthesise findings across these domains.

## Methods

### Protocol and registration

This scoping review was conducted in accordance with the Preferred Reporting Items for Systematic Reviews and Meta-Analyses extension for Scoping Reviews (PRISMA-ScR) [15] and the methodological framework described by Arksey and O’Malley [16] and refined by Levac et al. [17]. The protocol was not prospectively registered.

### Eligibility criteria

Studies were eligible if they reported on MD-ACL in human subjects and included clinical, radiological, histopathological, operative, or outcome data. Case reports, case series, cross-sectional studies, cohort studies, comparative studies, controlled trials, technical notes, systematic reviews, and narrative reviews were considered. Secondary evidence sources were retained to map the breadth and evolution of the field, but primary clinical and imaging studies were prioritised when summarising patient characteristics, imaging features, and treatment outcomes. To avoid double-counting, primary studies captured within the three included systematic reviews [7–9] were charted individually from their original publications rather than from the reviews’ summary data; the systematic reviews themselves were retained for their synthesis and methodological contribution but their pooled outcome figures were not combined with the individually charted primary studies.

Exclusion criteria were studies not available in English, conference abstracts without full-text availability, studies addressing posterior cruciate ligament mucoid degeneration without ACL-specific data, studies on isolated intraligamentous ganglion cysts without discussion of mucoid degeneration, and cadaveric or animal studies without clinical correlation.

### Information sources and search strategy

A systematic literature search was performed in PubMed/MEDLINE and Embase on 31 January 2026, covering records from database inception to that date. The core search combined terms for mucoid degeneration and the ACL: (“mucoid degeneration” OR “mucinous degeneration” OR “myxoid degeneration” OR “mucoid hypertrophy” OR “mucoid cystic degeneration”) AND (“anterior cruciate ligament” OR “ACL”). In PubMed/MEDLINE, title/abstract keywords were combined with the MeSH term “Anterior Cruciate Ligament”; in Embase, the equivalent Emtree term “anterior cruciate ligament” was exploded and combined with title/abstract/keyword free-text terms. No study-design or publication-type filters were applied at the search stage in order to maximise sensitivity, and no language restriction was applied until the eligibility-screening stage. The search yielded 117 records from PubMed/MEDLINE and 134 from Embase (251 records in total before de-duplication). The full electronic search strategy for each database, including controlled vocabulary terms, free-text terms, field tags, and date limits, is provided in Additional file 1. PubMed/MEDLINE and Embase were selected as the two databases with the broadest combined coverage of the orthopaedic and radiological literature relevant to this topic; Scopus and Web of Science were not searched separately given their substantial indexing overlap with Embase for this subject area, and the Cochrane Library was not searched given the near-total absence of randomised evidence in this field, reflected in the identification of only a single, non-randomised controlled trial across the entire body of included literature. These choices are acknowledged as a limitation in the Discussion. No additional citation tracking (backward or forward reference screening) or manual hand-searching of reference lists was performed beyond the electronic database searches described above. No dedicated grey-literature sources, conference-proceedings databases, or trial registries were searched; conference abstracts without an available full text were excluded at eligibility screening because they did not provide sufficient information for detailed charting. No health sciences librarian or information specialist was formally consulted in developing the search strategy; the strategy was instead developed independently by two authors (TV, WP) and cross-checked against the terminology used in all previously published reviews on this topic.

### Study selection and data charting

TV and WP independently screened titles, abstracts, and full texts. IG verified screening decisions, and any disagreements were resolved by consensus discussion among all three reviewers. TV and WP then charted the data independently and in duplicate, with discrepancies resolved by consensus discussion among all authors, and KV cross-checked the charted data against the source publications. Charting used a standardised form that captured publication year, country, study design, sample size, demographics, diagnostic definition, clinical presentation, MRI and arthroscopic findings, histology, associated intra-articular pathology, intervention, follow-up duration, outcome measures, postoperative laxity or instability, recurrence, and key conclusions.

### Synthesis approach

Given the heterogeneity of study designs, populations, interventions, and outcome measures, findings were synthesised narratively rather than pooled quantitatively. Evidence was organised thematically across the predefined domains of the review question. No formal risk-of-bias tool was applied, consistent with scoping review methodology; however, study design and overall methodological limitations were considered when interpreting the strength of inferences. Where the Discussion characterises a conclusion as strongly, moderately, or weakly supported, this reflects a narrative judgement based on study design, independence and consistency of findings across studies, sample size, and the presence or absence of a comparator group or longitudinal data; this judgement does not constitute a formal risk-of-bias or certainty-of-evidence assessment such as the Grading of Recommendations, Assessment, Development and Evaluations (GRADE) approach. The completed PRISMA-ScR checklist is provided as Additional file 2. An artificial-intelligence-based language tool was used during manuscript preparation to support language editing and formatting checks only; it was not used to identify, select, chart, or interpret evidence, and the authors verified all content. To orient the reader, findings are presented below following the review’s predefined conceptual domains: definition and terminology, epidemiology, pathophysiology, histopathology and molecular biology, clinical presentation, imaging findings, differential diagnosis, associated intra-articular pathology, morphometric risk factors, arthroscopic findings, management, and clinical outcomes.

## Results

**Fig. 1:**
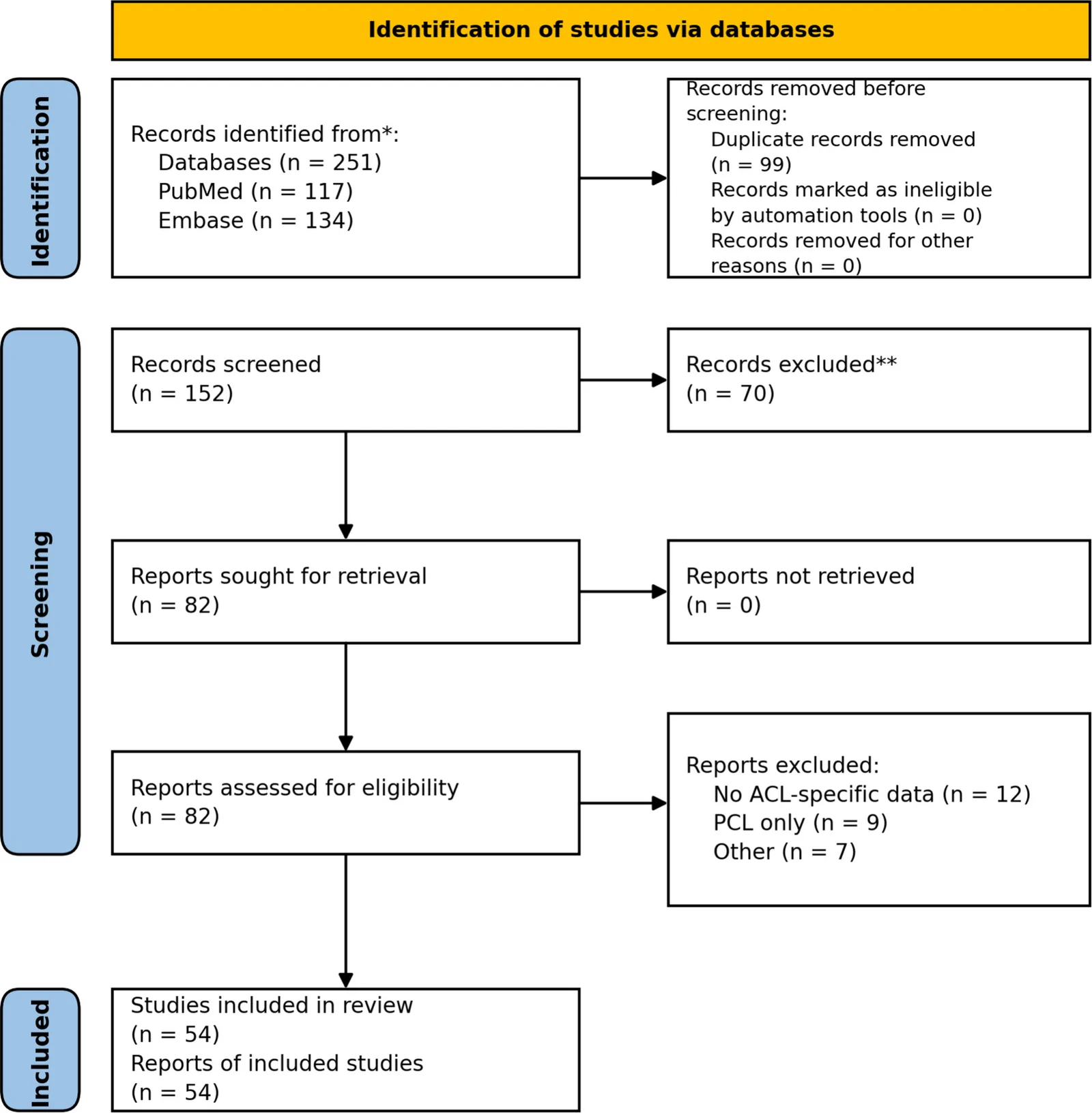
PRISMA-ScR flow diagram of study selection. A total of 251 records were identified through database searching. After removal of 99 duplicates, 152 records were screened by title and abstract. Eighty-two full-text articles were assessed for eligibility, and 54 publications were included in the final synthesis

The literature search identified 251 records. After removal of 99 duplicates, 152 records were screened by title and abstract. Eighty-two full-text articles were assessed for eligibility, and 54 publications were included in the final synthesis (Fig. 1). Included sources comprised case reports (*n* = 7), case series (*n* = 12), retrospective cohort or comparative studies (*n* = 20), prospective cohort studies (*n* = 3), systematic reviews (*n* = 3), one controlled clinical trial, technical notes (*n* = 5), and narrative reviews (*n* = 3). Overall, the primary clinical literature consisted mainly of retrospective case series and comparative observational studies. A summary of the mapped evidence is provided in Table 1, and detailed charting of selected primary studies is provided in Table 2. Studies were selected for detailed charting in Table 2 if they represented the largest cohort, the longest follow-up, or a landmark methodological or conceptual contribution (for example, the first clinical description, the defining MRI criteria, the only controlled trial, or the most recent histopathological data) within their evidence category or thematic domain; the remaining included sources are represented in the narrative synthesis and, in aggregate, in Table 1.

**Table 1:** Overview of mapped evidence included in the scoping review

Evidence category	Number of sources	Typical contribution	Main limitation
Case reports	7	Initial descriptions, unusual presentations, bilateral disease, younger patients	Very small numbers; limited generalisability
Case series	12	Clinical presentation, arthroscopic findings, early outcomes	Retrospective design; inconsistent PROMs
Retrospective cohort/comparative studies	20	Imaging discrimination, morphometric associations, treatment comparisons	Selection bias; confounding by concomitant pathology
Prospective cohort studies	3	Longitudinal imaging and arthroplasty-related outcomes	Few studies; still observational
Controlled clinical trial	1	Partial versus complete resection comparison	Small sample size; non-randomised allocation
Systematic reviews	3	Synthesis of management outcomes, debridement technique, and morphometric associations	Dependent on heterogeneous primary literature
Technical notes	5	Stepwise arthroscopic technique descriptions, operative pearls, and fibre-preserving decompression strategies	No comparative data, minimal or no outcome reporting, low evidentiary weight
Narrative reviews	3	Conceptual overviews of terminology, pathophysiology, imaging, and management	Non-systematic synthesis, risk of selection bias, dependent on limited and heterogeneous primary literature

**Table 2:** Detailed charting of selected key included sources in the synthesis

Study	Design	n	Intervention/assessment	Follow-up	Main contribution/key finding
Kumar et al. 1999 [2]	Case report	1	Arthroscopy and histology	NR	First formal clinical description of mucoid cystic degeneration causing posterior knee pain and terminal motion loss without instability
Bergin et al. 2004 [3]	MRI cohort	4221 MRIs; 44 with MD-ACL (18 isolated)	Retrospective MRI review	Cross-sectional	Defined widely used MRI criteria and showed coexistence with ACL ganglia
Cha et al. 2013 [18]	Surgical case series	68 knees	Arthroscopic treatment	Median 22 months (range, 12–120)	Characterised symptomatic presentation, including posterior knee pain (75%) and pain on terminal extension (91%)
Jung et al. 2012 [19]	Retrospective cohort	84 knees	MRI and arthroscopy correlation	Cross-sectional	Linked MD-ACL with increased posterior tibial slope and frequent concomitant chondral and meniscal lesions
Kwee et al. 2015 [20]	Radiological cohort	36 MD-ACL cases; 72 controls	Routine MRI cohort analysis	Cross-sectional	Showed strong association between MD-ACL and medial meniscal/cartilage damage
Kwee et al. 2018 [12]	Prospective cohort (OAI)	600 participants	MRI and radiographic follow-up	48 months	Associated MD-ACL with medial tibiofemoral joint-space narrowing progression, significant only in knees with less baseline cartilage damage
Hotchen et al. 2019 [14]	Comparative cohort	31 patients	Conservative vs arthroscopic management	Mean 4.0 years (range, 2.0–6.0) (conservative); mean 2.8 years (range, 2.0–3.6) (surgical)	Reported inferior pain and PROMs with conservative care compared with debridement
Celikyay et al. 2020 [4]	Comparative MRI study	43 MD-ACL; 37 complete ACL tears	MRI feature comparison	Cross-sectional	Celery stalk sign highly specific; fibre discontinuity strongly favoured tear
Gersing et al. 2021 [11]	Prospective cohort (OAI)	1899 right knees screened; 37 MD-ACL	MRI cartilage T2 progression	96 months	ACL abnormalities, including MD-ACL, were associated with accelerated cartilage matrix degeneration over 96 months
Lee et al. 2021 [21]	Retrospective surgical series	42 patients	Partial resection plus notchplasty	Mean 14.4 months (range, 12–106 months)	Good clinical outcomes with low reported recurrence after arthroscopic treatment
Rajani et al. 2022 [22]	Prospective multicentre study	442 UKA patients	Debulking vs no debulking during UKA	Minimum 2 years	Debulking associated with better function, range of motion, rest pain, and satisfaction in the UKA setting
Cilengir et al. 2023 [23]	Case–control MRI study	77 MD-ACL; 77 sprains	Ancillary MRI feature analysis	Cross-sectional	Tibial intraosseous cysts strongly increased probability of MD-ACL; decision-tree accuracy 79.2%
Oehler et al. 2023 [24]	Controlled clinical trial (non-randomised)	20 patients	Partial vs complete resection	Mean 16.8 months (range, 12–41)	Similar pain relief in both groups, but Lachman positivity was higher after complete resection (100% vs 20%)
Haidar et al. 2023 [13]	Retrospective series	60 patients	Arthroscopic partial resection	Mean 83.6 ± 44.8 months	Longest follow-up series; 71% patient satisfaction with generally durable symptom improvement, although 6 of the 51 patients available at final follow-up (11.7%) later underwent ACL reconstruction
Luo et al. 2024 [25]	Retrospective cohort	18 knees (16 conservative, 2 debridement)	Conservative management	Mean 21.8 months (range, 18–26)	Meaningful but incomplete symptom relief; worse outcomes with older age, longer duration, and meniscal tears
Choukimath et al. 2024 [26]	Technical note	—	Systematic arthroscopic debulking technique	—	Detailed stepwise operative technique for fibre-preserving decompression
Okazaki et al. 2026 [10]	Histopathology and transcriptomics	10 MD-ACL; 3 controls	Surgical tissue analysis	Cross-sectional	Demonstrated active matrix remodelling and differential gene-expression pathways in MD-ACL

*Abbreviations*: *ACL* Anterior cruciate ligament, *MD-ACL* Mucoid degeneration of the anterior cruciate ligament, *MRI* Magnetic resonance imaging, *NR* Not reported, *OAI* Osteoarthritis Initiative, *PROMs* Patient-reported outcome measures, *UKA* Unicompartmental knee arthroplasty

### Definition and terminology

The accepted current term is mucoid degeneration of the ACL, reflecting diffuse interstitial accumulation of glycosaminoglycan-rich mucinous material within the substance of an intact ligament. This terminology has evolved chronologically as understanding of the condition matured: the earliest reports used mucoid cystic degeneration in 1999 [2], reflecting an initial conflation with cystic lesions; mucoid hypertrophy [27] later emphasised the macroscopic ligament enlargement; and mucoid metaplastic degeneration [28] emphasised a presumed tissue-transformation mechanism, both terms dating from 2008. The terms mucinous degeneration and myxoid degeneration are also used interchangeably. This terminological heterogeneity complicates cross-study comparison and partly explains variation in reported prevalence and diagnostic thresholds [6].

MD-ACL should be distinguished from two entities with which it may coexist. An intraligamentous ganglion cyst is a focal, encapsulated, fluid-filled structure with defined margins, whereas MD-ACL is a diffuse, interstitial process without a true cystic envelope [3]. A partial ACL tear may show focal fibre disruption or attenuation with partial preservation of overall continuity and orientation; a history of trauma and clinical laxity may support the diagnosis but are not invariably present, and neither feature is characteristic of uncomplicated MD-ACL [4–6]. Ganglion cysts and MD-ACL coexisted in 26 of the 74 affected knees (35%) in that series [3]. The diagnostic criteria proposed across clinical, imaging, arthroscopic, and histopathological modalities are summarised in Table 3, illustrating how the definition of MD-ACL has been operationalised differently across the literature.

**Table 3:** Evolution of diagnostic definitions and associated morphometric or molecular findings for MD-ACL

Modality/source	Reported definition, criterion, or associated finding	Reported role or performance
Clinical/histological (Kumar et al. 1999) [2]	Arthroscopic identification of intact, mucin-infiltrated ACL fibres with histological confirmation of mucoid material; absence of clinical instability	First formal diagnostic description; established MD-ACL as a distinct entity from ligament tear
MRI (Bergin et al. 2004) [3]	Increased intraligamentous signal with preserved bundle orientation and continuity; poorer delineation of individual bundles on T1-weighted than fluid-sensitive sequences	Most widely adopted MRI criteria in subsequent literature
MRI – celery stalk sign (Celikyay et al. 2020) [4]	Diffuse intraligamentous high signal traversed by preserved low-signal longitudinal fibres	Present in ~ 66% of MD-ACL cases; highly specific when present, but absence does not exclude diagnosis
MRI – ancillary features (Cilengir et al. 2023) [23]	Tibial intraosseous cysts, ACL thickening, absence of bone contusion or anterior tibial translation	Decision-tree model combining ancillary features achieved 79.2% diagnostic accuracy versus sprain
MRI – associated morphometric finding, not a diagnostic criterion (Kim et al. 2023) [29]	Posterior tibial slope > 12.2° (AUC = 0.765); notch width index ≤ 28.27% (AUC = 0.849); transverse notch angle ≤ 47.4°	Proposed adjunctive radiographic risk thresholds; not yet externally validated
Arthroscopic (Narvekar & Gajjar 2004; Choukimath et al. 2024) [26, 30]	Loss of synovial lining, continuous ACL fibres, increased ACL volume, yellowish discolouration, extrusion of mucinous material on probing	Confirms MRI diagnosis intraoperatively; requires direct visualisation
Histopathological/molecular (Okazaki et al. 2026) [10]	Alcian-blue-positive glycosaminoglycan accumulation, disorganised collagen architecture, reduced type I collagen, differential expression of matrix and signalling genes	Provides tissue-level confirmation after surgical sampling; not a routinely required clinical diagnostic standard, and current use is limited to research settings

Reference numbers correspond to the main reference list. The morphometric and molecular rows describe findings associated with MD-ACL rather than criteria required for diagnosis; core diagnostic definitions are clinical/histological, MRI, and arthroscopic

*ACL* Anterior cruciate ligament, *AUC* Area under the curve, *MD-ACL* Mucoid degeneration of the anterior cruciate ligament, *MRI* Magnetic resonance imaging

### Epidemiology

Reported prevalence varies according to diagnostic definition, MRI field strength, and whether ganglion cysts and MD-ACL are analysed together. In the seminal MRI series by Bergin et al., 74 of 4221 examinations met imaging criteria for either ACL ganglion or mucoid degeneration; mucoid degeneration was present in 44 examinations, in 18 of which it occurred in isolation, while 56 showed discrete ganglia and 26 showed features of both [3]. Salvati et al. reported MD-ACL in 64 of 1215 examinations (5.3%) [28], and routine MRI cohort data suggest that MD-ACL is identified more often at 3 T than at 1.5 T, indicating that field strength influences conspicuity and reported prevalence [20].

MD-ACL predominantly affects middle-aged adults. In the combined ganglion and mucoid-degeneration cohort of Bergin et al., the mean age was 42 years with no clear sex predilection [3], while Kwee et al. found a median age of 55.5 years in a routine MRI cohort and Cha et al. reported a median age of 51 years in a symptomatic surgical series [18, 20]. Across contemporary cohorts, patients with MD-ACL are older than those with normal or torn ACLs, and diagnoses are made more often at 3 T than at 1.5 T [4, 20]. Bilateral symptomatic involvement remains rare [31].

Overall, the available evidence suggests that clinically recognised MD-ACL on routine MRI is uncommon but not exceptional. Recognition has probably improved as radiologists have applied more consistent criteria and more actively distinguished MD-ACL from partial tearing [3, 5, 6].

### Pathophysiology

The aetiopathogenesis of MD-ACL is multifactorial and remains incompletely understood. Four non-mutually exclusive mechanisms recur across the literature: synovial disruption, repetitive mechanical microtrauma, degeneration associated with medial compartment osteoarthritis, and impaired matrix turnover with aggrecan accumulation [6, 32].

The synovial theory proposes that disruption of the synovial sheath investing the ACL allows joint fluid to permeate the ligament substance [6]. Recent histopathological observations are consistent with a possible protective role of the ACL synovium, and congenital or acquired displacement of synovial tissue into the ligament has also been proposed [10].

The traumatic and mechanical theory is the most consistently supported in recent studies. Increased posterior tibial slope promotes anterior tibial translation and repetitive loading, while a narrow intercondylar notch width index may increase impingement of a hypertrophied ACL against the notch roof or lateral wall during terminal extension [10, 19, 29]. In older patients, MD-ACL also appears to overlap with a broader degenerative phenotype involving medial meniscal damage and medial compartment cartilage pathology [10, 11].

A more recent mechanistic framework centres on accumulation of denatured aggrecan within the ligament interstitium, arising from imbalance between matrix synthesis and lymphatic clearance [32]. This hypothesis remains exploratory, but it offers a biologically coherent model that may reconcile ligament swelling, altered matrix composition, and the coexistence of mechanical and degenerative pathways.

### Histopathology and molecular biology

Histologically, MD-ACL is characterised by diffuse accumulation of glycosaminoglycan-rich mucinous material, typically Alcian blue positive, dissecting between intact but thinned and displaced collagen fibres [6, 10]. The collagen fibres remain structurally continuous, and there is no true cystic wall. Focal neovascularisation, haemosiderin deposition, and occasional chondroid metaplasia may also be seen [6, 18]. The synovial lining is absent or atrophic in many histologically confirmed cases and may correlate with greater degeneration severity [6, 28, 33].

In the largest histopathological and transcriptional study to date, ten surgically confirmed MD-ACL samples were compared with three normal cadaveric controls [10]. The investigators found disorganised collagen architecture, reduced type I collagen, increased cellularity, abundant glycosaminoglycan, angiogenesis, chondrogenesis, and occasional lipid deposition. NanoString analysis identified 155 differentially expressed genes, with upregulation of extracellular matrix, collagen biosynthesis, focal adhesion kinase, platelet-derived growth factor signalling, and PI3K-Akt pathways [10]. These data support an active remodelling process rather than passive degeneration alone.

Makino et al. also reported MRI-arthroscopic-histological correlation in a surgical series, supporting the pathological validity of the imaging findings described in earlier MRI studies [34].

### Clinical presentation

MD-ACL typically presents as insidious, progressive knee pain of variable duration, ranging from weeks to years [4, 6]. The most consistent symptom is posterior or deep central knee pain, reported in 24 of 31 patients (77%) in a clinical cohort [14] and in 51 of 68 knees (75%) in a symptomatic surgical series [18]. Pain is usually exacerbated by terminal flexion or extension. A bounce home–like phenomenon, defined as painful resistance to full passive extension, was reported in 77 of 84 knees (91.7%) by Jung et al. [19], while Cha et al. documented pain on terminal extension in 62 of 68 knees (91%) [18]. Limitation of terminal flexion, often to around 100°, affects about half of symptomatic patients [6, 14]. Individual case reports illustrate the same pattern, including presentation with posterior knee pain and painful deep flexion in the absence of instability [35] and, rarely, simultaneous mucoid degeneration of both cruciate ligaments presenting as a refractory flexion contracture after two non-diagnostic arthroscopies [36].

Clinical examination usually demonstrates a stable knee. The anterior drawer, Lachman, and pivot-shift tests are generally negative, with a firm Lachman end point suggesting preserved ligament continuity [4, 6]. Mild effusion may be present, and meniscal provocation tests may be positive where concomitant meniscal pathology coexists. Standard radiographs are often unremarkable apart from early osteoarthritic change or notch narrowing in older patients [6].

Symptoms may persist despite non-operative treatment. In the only comparative study of conservative versus surgical management, 14 of 18 conservatively managed patients available for follow-up (77.8%) still reported knee pain and regular analgesic use, compared with none of the seven patients treated by arthroscopic debridement [14]. Luo et al. nevertheless showed that selected patients may improve with conservative management, although symptom relief was incomplete and outcomes were worse in older patients, those with longer symptom duration, and those with concomitant meniscal tears [25].

### Imaging findings

MRI is the principal imaging modality for MD-ACL. The diagnostic criteria described by Bergin et al. include increased ACL signal, preserved overall orientation and continuity, and poorer delineation of individual bundles on T1-weighted than on fluid-sensitive sequences [3]. Signal intensity remains lower than free synovial fluid, which helps distinguish diffuse mucoid change from a focal intraligamentous cyst [6].

The celery stalk sign, defined as diffuse intraligamentous high signal traversed by preserved low-signal longitudinal fibres, is the most characteristic MRI finding [4]. In a comparative study of 43 MD-ACL and 37 complete ACL tears, the sign was present in approximately 66% of MD-ACL cases and was highly specific when present [4]. Its absence, however, does not exclude the diagnosis. ACL thickening, often with a cross-sectional diameter greater than 10 mm, is another useful discriminating feature, as are associated peri-ACL ganglion cysts, intraosseous cysts, and absence of the bone contusions and anterior tibial translation typically seen in acute tears [4, 23].

Recent observational studies suggest that ACL abnormalities, including MD-ACL, are associated with accelerated structural cartilage deterioration, particularly in the medial compartment [11, 12]. These findings support an association with osteoarthritic progression, but they do not establish a direct causal relationship.

**Fig. 2:**
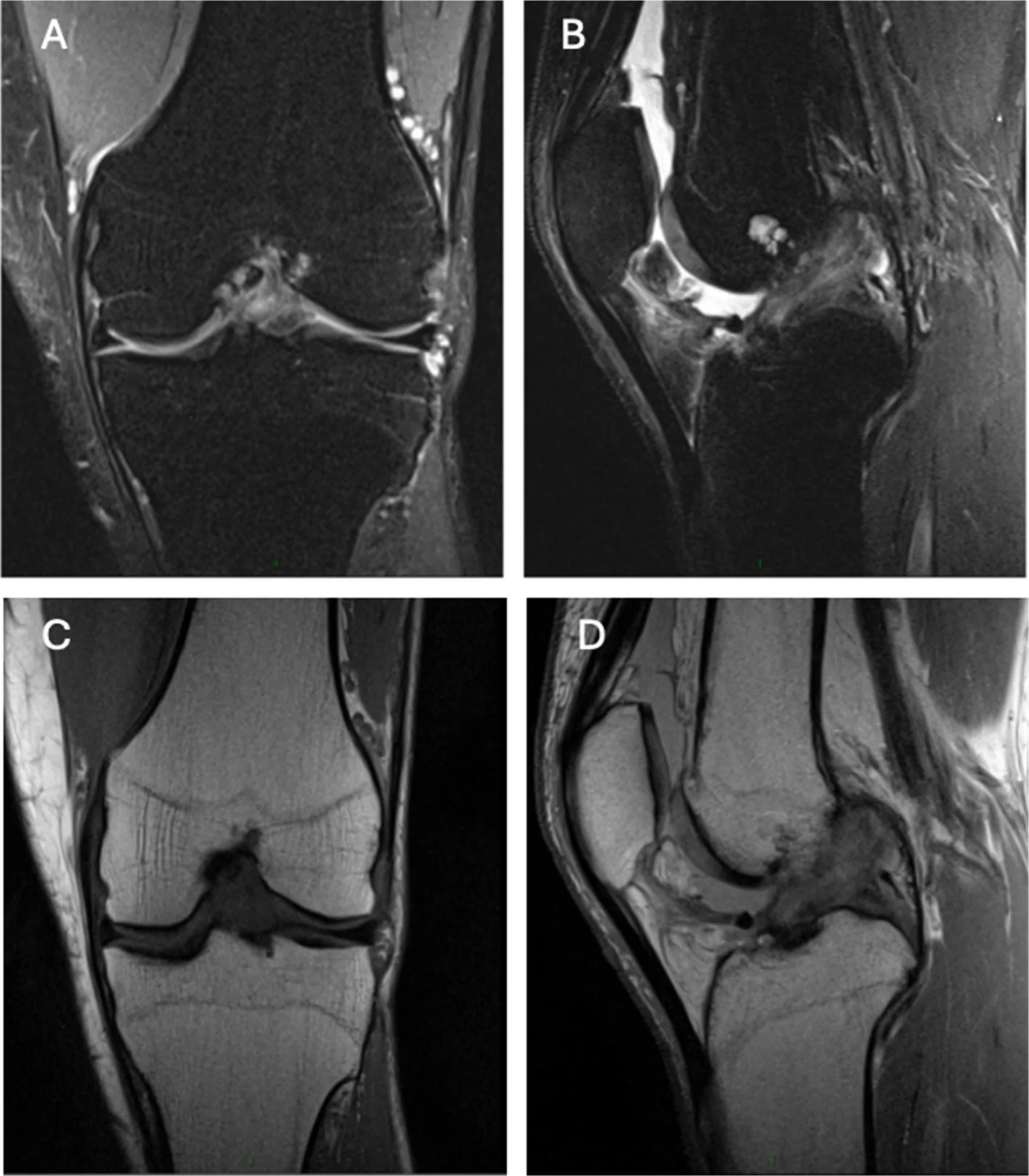
Representative magnetic resonance imaging findings of mucoid degeneration of the anterior cruciate ligament. **A** Coronal T2-weighted image demonstrating diffuse ACL thickening with increased intraligamentous signal. **B** Sagittal T2-weighted image showing the characteristic celery stalk sign, with longitudinal low-signal fibres traversing high-signal mucoid material. **C** Coronal T1-weighted image demonstrating diffuse enlargement of the ACL with intermediate intraligamentous signal intensity. **D** Sagittal T2-weighted image illustrating diffuse high signal within the thickened ACL substance consistent with mucoid infiltration

Representative MRI findings of MD-ACL, including diffuse ligament thickening, increased intraligamentous signal, and the celery stalk sign, are illustrated in Fig. 2.

### Differential diagnosis

The most important differential diagnosis is ACL tearing. Complete tears are usually distinguished by fibre discontinuity, altered ligament orientation, bone contusions, and anterior tibial translation [4]. Partial tears are more challenging because they may preserve apparent continuity; in practice, the combination of trauma history, clinical laxity, and demonstrable fibre disruption remains central to differentiation [4–6]. Integration of MRI findings with the trauma history and clinical stability assessment generally improves diagnostic confidence, and this ambiguity arises chiefly when MRI is read without that clinical context.

Intraligamentous ganglion cysts show focal, lobulated, fluid-equivalent signal with mass effect on ACL fibres rather than the diffuse interstitial hyperintensity of MD-ACL [3]. Cyclops lesions and arthrofibrosis usually present as anterior notch masses rather than diffuse intraligamentous change, and are typically associated with prior ACL reconstruction. Synovial proliferative lesions and other unusual notch lesions should be considered when morphology is atypical. Table 4 summarises the differentiating imaging and clinical features of MD-ACL, intraligamentous ganglion cyst, partial ACL tear, and complete ACL tear.

**Table 4:** MRI and clinical features differentiating MD-ACL from ganglion cyst, partial tear, and complete tear

Feature	MD-ACL	Intraligamentous ganglion cyst	Partial ACL tear	Complete ACL tear
Fibre continuity	Preserved, diffusely abnormal	Preserved, focal mass effect on fibres	Partial preservation with focal disruption or attenuation	Discontinuous (97% of tears) [4]
Signal pattern	Diffuse intraligamentous signal, intermediate on T1, high on fluid-sensitive sequences	Focal, fluid-equivalent signal, higher than MD-ACL	Focal abnormal signal at the tear site; orientation may be partly preserved	Diffuse abnormal signal with loss of normal fibre orientation
Celery stalk sign	Present in 66% of cases [4]	Absent	Not established in the included literature	Absent (0% in complete tears) [4]
Ligament thickening	Present in 100% of cases [4]	Focal expansion only	Variable; not systematically reported	Uncommon (19% of tears) [4]
Ganglion cysts/intraosseous cysts	Ganglion cysts 70%; intraosseous cysts 63% [4]	Defining feature by definition	Not established in the included literature	Ganglion cysts 14%; intraosseous cysts 8% [4]
Bone contusion/anterior tibial translation	Rare (contusion 2%; ATT 14%) [4]	Absent	May be present, particularly with trauma	Common (contusion 65%; ATT 70%) [4]
Clinical correlate	Stable knee; chronic posterior or deep central pain	Often incidental and asymptomatic	Trauma history and clinical laxity may be present but are not universal	Trauma history, laxity, positive Lachman/anterior drawer/pivot-shift
Reported diagnostic performance	Ancillary-feature decision tree: 79.2% accuracy vs sprain [23]	Not systematically reported	Cilengir et al.’s ancillary-feature decision tree (79.2% accuracy, see MD-ACL column) was developed and evaluated against a comparator termed “ACL sprain”; the exact tear-grade composition of that comparator was not fully specified	ACL angle ≤ 36°: 78% sensitivity, 91% specificity for tear [4]

Percentages for MD-ACL and complete ACL tear are from a single retrospective comparative study (43 MD-ACL cases vs 37 complete ACL tears) [4]; no study in the included literature used the same feature set to compare MD-ACL against complete and partial tears simultaneously; the partial-tear performance data (Cilengir et al.) were derived against a comparator that the source study terms “ACL sprain”, illustrated there with partial-tear examples, but the exact tear-grade composition of that comparator was not fully specified, so this estimate should be read as indirectly relevant to the partial-tear differential rather than as validated performance data. In clinical practice, this differential is usually resolved through trauma history and examination rather than MRI appearance alone, and is most likely to present a diagnostic challenge when imaging is interpreted in isolation

*ACL* Anterior cruciate ligament, *ATT* Aterior tibial translation, *MD-ACL* Mucoid degeneration of the anterior cruciate ligament, *MRI* Magnetic resonance imaging

### Associated intra-articular pathology

MD-ACL is consistently associated with concomitant intra-articular pathology. In the Sweed et al. systematic review, 82% of arthroscopically treated patients had chondral lesions and 69% had meniscal tears [7]. In a series of 84 arthroscopically treated knees, Jung et al. documented medial meniscal tears in 44.0%, lateral meniscal tears in 13.1%, and medial femorotibial chondral lesions in 67.9%, of which 38 of 57 were Outerbridge grade III or IV [19]. Kwee et al. demonstrated that severe medial tibiofemoral cartilage damage was independently associated with MD-ACL in a radiological cohort [20].

### Morphometric risk factors

An increased posterior tibial slope is the most consistently replicated morphometric association with MD-ACL [19, 29, 37]. Kim et al. identified a posterior tibial slope threshold of greater than 12.2° (area under the curve [AUC] = 0.765) and a notch width index threshold of 28.27% or less (AUC = 0.849), with a transverse notch angle of 47.4° or less also associated with MD-ACL [29]. A systematic review by Vivekanantha et al. of seven studies likewise identified increased posterior tibial slope, reduced notch width index, and greater tibial tuberosity-trochlear groove distance as the morphometric factors most consistently associated with MD-ACL [9]. This association is not universal: in a case–control study of 20 knees with MD-ACL, 66 with ACL tears, and 86 matched controls, Ravi et al. found no difference in medial, lateral, or coronal tibial slope between MD-ACL and intact ACLs, and instead reported higher body mass index and older age in the MD-ACL group [38]. Findings on notch morphology are similarly mixed: Hotchen et al. were unable to confirm a reduced notch width index in MD-ACL, and reported a higher mean index in knees with Kellgren–Lawrence grade 2–4 changes (0.273) than in knees without radiographic osteoarthritis (0.252), which they interpreted as distinguishing primary notch stenosis from secondary impingement by osteophytes [14]. A further comparative study of 67 patients with MD-ACL and 67 matched controls reproduced this pattern: the notch width index was lower overall in MD-ACL (0.26 versus 0.28) and the posterior tibial slope larger (11.3° versus 9.2°), but within the MD-ACL group the index was lower in knees without radiographic osteoarthritis (0.25) than in those with it (0.28), while the slope did not differ between these subgroups [39]. Additional reported associations include trochlear dysplasia, greater tibial tuberosity-trochlear groove distance, and increased lateral tibial slope [40, 41].

The clinical relevance of the posterior tibial slope association is further illustrated by reports of MD-ACL developing after opening wedge high tibial osteotomy when slope is excessively increased [42], and by a propensity score-matched study showing inferior intermediate-term outcomes when MD-ACL developed after osteotomy [43]. These findings are best interpreted as observational associations that may reflect cumulative biomechanical loading rather than proven causal risk factors.

### Arthroscopic findings

Arthroscopy confirms and complements the MRI diagnosis. Common findings include intact overall fibre architecture, diffuse ACL hypertrophy, yellowish or brownish discolouration, loss of the normal synovial covering, and extrusion of mucinous material on probing [4, 6, 26, 30]. Probing usually demonstrates preserved functional competence despite the abnormal appearance. Notch impingement during terminal extension is commonly visible intraoperatively [6, 19].

Because concomitant meniscal and chondral pathology is frequent, systematic inspection of all knee compartments remains important at the time of arthroscopy [7].

**Fig. 3:**
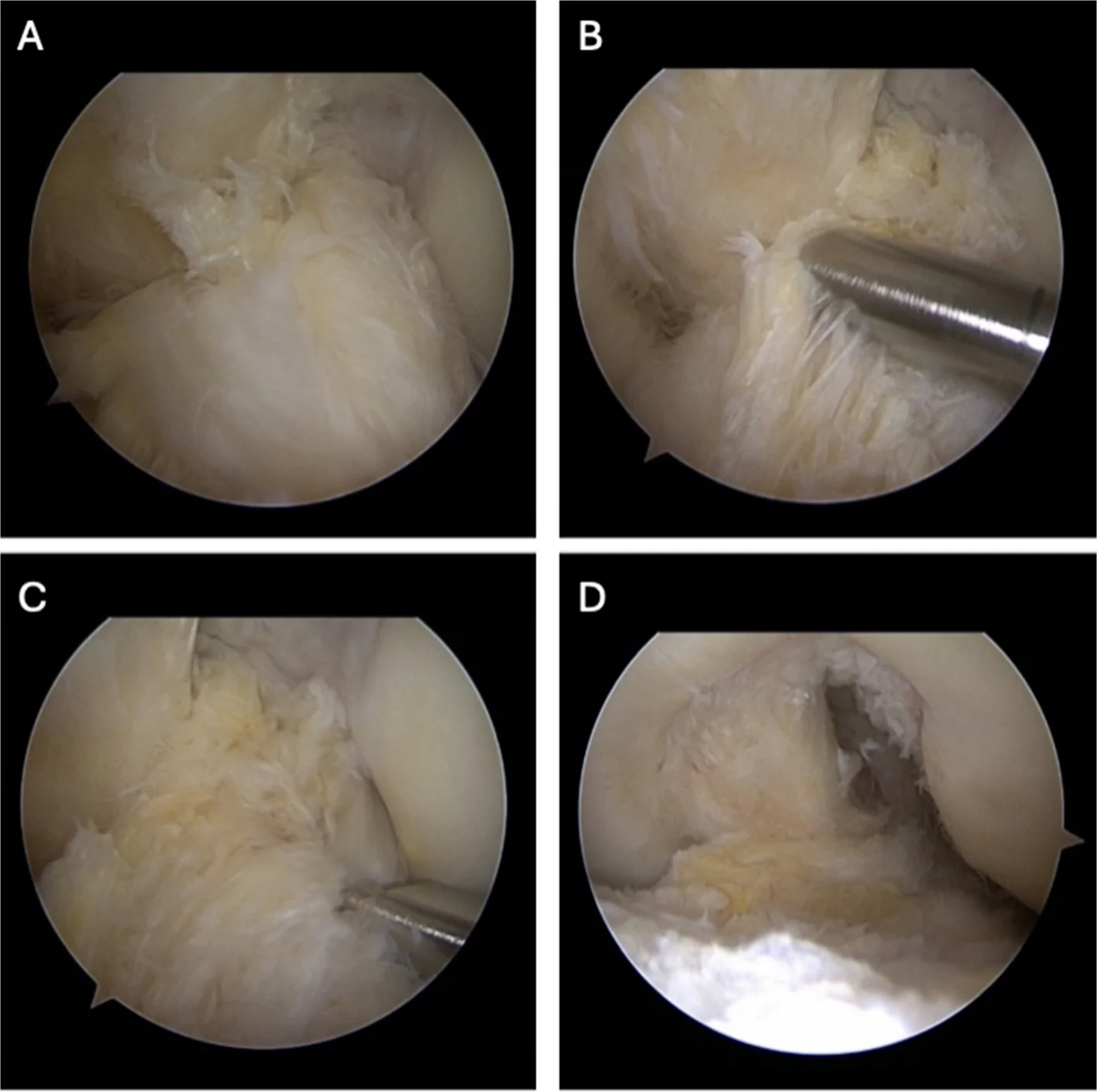
Representative arthroscopic appearances and debridement spectrum in mucoid degeneration of the anterior cruciate ligament. **A** Hypertrophied anterior cruciate ligament with yellow mucoid degeneration within the intercondylar notch. **B** Arthroscopic debridement in progress, with resection of mucoid tissue from the ligament substance. **C** Partially debrided anterior cruciate ligament showing preserved residual fibres with persisting yellow mucoid change. **D** Intercondylar notch after extensive resection in a selected case

Representative arthroscopic appearances of MD-ACL are illustrated in Fig. 3A and 3C. Figure 3A shows the untreated hypertrophied ligament, whereas Fig. 3C shows a partially debrided ligament with persisting yellow mucoid change.

### Management

Initial treatment generally comprises analgesia or non-steroidal anti-inflammatory medication, physiotherapy, and activity modification, particularly in patients without marked mechanical symptoms or with substantial coexistent osteoarthritis. The available evidence for conservative management is limited and suggests incomplete symptom relief in many patients [14, 25].

Arthroscopic debridement remains the most commonly reported operative treatment for persistent symptoms. The guiding principle is to remove enough ACL bulk to relieve notch impingement while preserving as many mechanically competent fibres as possible [6–8, 26]. Reported techniques include shaver or radiofrequency-assisted debulking, selective bundle treatment, notchplasty when structural impingement is present, and reduction plasty in fibre-preserving approaches [21, 26, 44–49]. Recent technical descriptions have addressed the notch and the ligament together, combining radiofrequency ablation with targeted notchplasty at defined anatomical sites [48], and describing bicompartmental decompression through anterior portals alone [49]. Representative stages of arthroscopic treatment are shown in Fig. 3B-D, including active debridement, partial fibre-preserving debridement, and more extensive resection in a selected case.

The main surgical controversy concerns extent of resection. Complete resection may decompress the ligament more thoroughly but increases postoperative laxity, whereas partial resection is generally favoured to reduce instability risk [7, 8, 24, 50]. In younger or athletically active patients, early discussion of possible ACL reconstruction is appropriate if extensive resection is anticipated or if the residual ligament is judged biomechanically insufficient [51–53]. Emerging strategies such as platelet-rich plasma augmentation and image-guided sleeve injection remain preliminary and should currently be regarded as exploratory rather than standard care [54–56].

In the arthroplasty setting, Rajani et al. reported in a prospective multicentre unicompartmental knee arthroplasty study (n = 442) that debulking of a mucoid ACL was associated with better Knee Society functional scores, International Knee Documentation Committee (IKDC) scores, range of motion, rest pain, and patient satisfaction at 2 years than no debulking [22]. This suggests that preoperative recognition of MD-ACL may influence arthroplasty planning and intraoperative strategy in selected patients.

### Clinical outcomes

Across published series, arthroscopic treatment generally improves pain and range of motion. In the Sweed et al. systematic review, postoperative pain relief was reported in 53.8% to 95% of patients, with improvement in range of motion and patient-reported outcome measures across most studies [7]. Haidar et al., reporting the longest follow-up series, found 71% patient satisfaction at a mean 83.6 months [13]. Interpretation of outcomes remains limited by heterogeneity in concomitant procedures, inconsistent instability assessment, and variable use of patient-reported outcome measures.

Postoperative laxity is common, but symptomatic instability is less frequent. In the Sweed review, a positive Lachman test was documented in 40% of assessed patients, whereas symptomatic instability occurred in 6% [7]. The instability risk appears highest when resection is extensive or complete [13, 24, 57, 58]. In the controlled trial by Oehler et al., complete resection resulted in universally positive Lachman tests compared with 20% after partial resection, despite similar pain relief and no patient in either group seeking reconstruction for symptomatic instability [24]. Recurrence after arthroscopic treatment is infrequently reported, although follow-up remains inconsistent across studies [21, 57].

## Discussion

The most important findings of this review are threefold. First, diagnosis of MD-ACL depends on integrating MRI features (preserved fibre continuity, ligament thickening, notch morphology) with a clinically stable knee on examination, rather than on any single sign in isolation [4, 6, 23]. The celery stalk sign is characteristic and highly specific when present, but it is not universal and should not be treated as a standalone diagnostic test [3, 4, 23]; arthroscopy can confirm the diagnosis and relieve impingement, but concomitant meniscal or chondral disease frequently complicates both preoperative attribution of symptoms and interpretation of postoperative improvement [7, 19, 20]. Second, recent histopathological and transcriptomic data reframe MD-ACL as an active tissue remodelling process, characterised by disorganised collagen architecture, increased cellularity, and upregulated matrix and signalling pathways, rather than a purely passive degenerative change, and this is complemented by longitudinal OAI imaging data that position MD-ACL within a broader degenerative knee phenotype [10–12]. Third, arthroscopic partial debridement generally relieves pain and improves motion, but the available evidence indicates that postoperative laxity becomes more likely as resection becomes more extensive, a trade-off that should directly inform preoperative counselling and the planned extent of resection [7, 13, 24, 58].

### Unresolved controversies

Two questions in particular are not resolved by the current literature. The first is whether MD-ACL should be regarded as a primary disease process or as a phenotypic marker of a broader degenerative process affecting the medial compartment. This relationship requires careful interpretation because studies at different stages of disease and using different sampling frames report divergent pictures of the association: symptomatic arthroscopic series often include patients without advanced radiographic osteoarthritis, whereas imaging-based cohorts and OAI analyses show consistent associations between MD-ACL and meniscal damage, cartilage loss, and structural progression [11, 12, 20]. These findings are not necessarily contradictory; they more plausibly reflect differences in study design, patient selection, and disease stage. At present, the literature supports a meaningful association between MD-ACL and degenerative knee pathology, but it does not establish that MD-ACL independently drives osteoarthritis progression. The second concerns posterior tibial slope, the most consistently replicated morphometric association with MD-ACL [19, 29, 37], although one case–control study found no slope difference between MD-ACL and intact ligaments [38]. Posterior tibial slope is a fixed skeletal parameter established during growth and does not itself change through a degenerative process; reports of MD-ACL developing after opening wedge high tibial osteotomy, when slope is iatrogenically increased, therefore provide circumstantial support for a possible load-related mechanism [42, 43], although a single case report and one propensity score-matched cohort cannot establish causality. The broader body of cross-sectional association studies likewise cannot confirm a causal role, since a correlation between congenital posterior tibial slope and MD-ACL could instead reflect confounding by other anatomical factors rather than a direct biomechanical effect. Resolving both questions would require prospective cohorts that track morphometric and imaging findings longitudinally against incident MD-ACL and structural progression.

### Strength of the evidence

The strength of the underlying evidence varies considerably across these conclusions. The MRI diagnostic criteria and the association with concomitant meniscal and chondral pathology are supported by consistent findings across multiple independent cohorts and represent the most robust conclusions of this review [3, 4, 7, 19, 20]. The association between more extensive resection and postoperative laxity is supported by one non-randomised controlled trial and several concordant case series and can be considered moderately robust [7, 13, 24]. In contrast, the causal status of the posterior tibial slope association, the independent contribution of MD-ACL to osteoarthritis progression, and the benefit of emerging treatments such as platelet-rich plasma augmentation remain weakly supported, resting on small, uncontrolled, or purely observational data, and should currently be regarded as hypotheses rather than established conclusions [32, 42, 43, 54–56]. The evidence on conservative management also warrants caution: only one comparative study and one small, uncontrolled cohort have addressed non-operative treatment to date. The comparative study reported persistent pain and analgesic use in most conservatively managed patients, and the newer series, while showing that selected patients improve, included no comparator arm [14, 25]. This limited evidence base is not sufficient to substantially change current treatment paradigms, and conservative management should continue to be regarded as a reasonable initial option in appropriately selected patients rather than as an evidence-supported alternative to arthroscopic treatment.

### Management implications

Management should be individualised on the basis of the evidence above. Arthroscopic partial debridement, with or without notchplasty, offers symptom relief for many patients, particularly when notch impingement is the dominant problem, but the trade-off with postoperative laxity means that younger or athletically active patients may warrant earlier discussion of reconstruction when major resection is anticipated or residual ligament competence is doubtful [51–53]. A suggested management approach synthesising these considerations by clinical scenario is presented in Table 5.

**Table 5:** Suggested management approach by clinical and disease characteristics

Clinical scenario	Potential management approach	Supporting rationale	Key caveat
Mild symptoms, minimal notch impingement, lower-demand patient	Analgesia/NSAIDs, physiotherapy, activity modification	Reasonable first-line option in appropriately selected patients	Evidence is limited; most conservatively managed patients in the only comparative study still reported pain at follow-up [14, 25]
Persistent symptoms after conservative trial, notch impingement dominant, stable knee	Arthroscopic partial debridement ± notchplasty	Most commonly reported operative treatment; relieves impingement while limiting laxity risk [7, 8, 21, 26, 44–47]	Preserve maximal mechanically competent fibre; reassess extension/flexion intraoperatively
Extensive ligament involvement or advanced degeneration, laxity risk acceptable	Complete resection	Decompresses the ligament more thoroughly; may be considered in selected lower-demand patients when adequate decompression cannot be achieved while preserving competent fibres [24, 50]	Higher postoperative laxity risk (positive Lachman up to 100% vs 20% after partial resection) [24]
Younger or athletically active patient; major resection anticipated or residual fibre competence doubtful	Early discussion of ACL reconstruction	May avoid delayed instability in high-demand patients [51–53]	Indications and timing for reconstruction are not standardised in the current literature
Concomitant meniscal or chondral pathology	Assess systematically and treat according to standard indications	Coexists in a large proportion of surgical series (up to 82% chondral, 69% meniscal) [7, 19]	Systematic inspection of all compartments is recommended regardless of primary indication
Incidental MD-ACL identified during unicompartmental knee arthroplasty planning	Consider concurrent debulking	Associated with better functional scores, range of motion, and satisfaction at 2 years in one prospective multicentre study [22]	Evidence limited to a single study; not yet replicated
Interest in emerging adjuncts (platelet-rich plasma, image-guided injection)	Regard as exploratory, not standard care	Preliminary evidence only, from small uncontrolled series [54–56]	May be considered only in selected circumstances or research settings; comparative effectiveness against established treatment is unknown

This table synthesises approaches reported across the included literature and does not constitute a validated clinical decision rule; management should be individualised as discussed in the text

*ACL* Anterior cruciate ligament, *MD-ACL* Mucoid degeneration of the anterior cruciate ligament, *NSAIDs* Non-steroidal anti-inflammatory drugs

### Research priorities

Rather than a general call for more prospective studies, three specific gaps should be prioritised. First, prospective cohorts that track posterior tibial slope and other morphometric risk factors from before MD-ACL onset are needed to clarify whether increased posterior tibial slope is a genuine causal risk factor for MD-ACL development, or whether the cross-sectional association instead reflects confounding by other anatomical factors. Second, comparative studies of conservative versus operative management, using standardised outcome measures, are needed before conservative treatment can be positioned confidently within a treatment algorithm. Third, replication of the transcriptomic and histopathological findings in larger, independent cohorts is needed to establish whether the proposed active-remodelling model of MD-ACL generalises beyond the initial surgical series in which it was described.

### Strengths and limitations

This review brings together clinical, imaging, arthroscopic, histopathological, and molecular evidence in one place and incorporates recent longitudinal and molecular studies that fall outside the search windows of earlier reviews. Its limitations mirror those of the underlying literature. Most included studies are retrospective single-centre series, imaging cohorts, or case reports; comparative data are scarce; validated outcome measures are used inconsistently; and definitions of MD-ACL remain heterogeneous across studies, which itself limits direct comparison. The protocol was not prospectively registered; although prospective registration is not mandatory for scoping reviews, methodological transparency was prioritised through adherence to the PRISMA-ScR reporting framework (Additional file 2) and full disclosure of the search strategy (Additional file 1). The search was restricted to PubMed/MEDLINE and Embase and to English-language publications, no additional citation tracking or hand-searching of reference lists was performed, and no health sciences librarian was formally consulted in developing the search strategy, although it was independently constructed and cross-checked by two authors (TV, WP) against the terminology used in all previously published reviews on this topic. No formal risk-of-bias or certainty-of-evidence appraisal was performed, consistent with scoping review methodology but nonetheless a constraint on the strength of the conclusions drawn here.

## Conclusions

MD-ACL is a recognised cause of atraumatic posterior knee pain and terminal motion restriction in patients who usually do not demonstrate clinical instability. Diagnosis relies mainly on MRI interpreted in the appropriate clinical context, with attention to preserved ligament continuity, ACL thickening, and ancillary imaging features rather than any single sign in isolation. Recent histopathological and transcriptomic evidence suggests that MD-ACL involves active matrix remodelling rather than passive mucin accumulation alone, while longitudinal associations with osteoarthritis should currently be interpreted as associative rather than causal.

When symptoms persist despite conservative management, arthroscopic partial debridement, with or without notchplasty, remains the most commonly reported operative treatment and usually improves pain and range of motion. Because postoperative laxity becomes more likely with more extensive resection, management should be individualised, particularly in younger and more active patients. Prospective comparative studies using standardised diagnostic definitions and outcome reporting are needed to guide treatment more definitively.

## Supplementary Information

Additional file 1. Full electronic search strategies. Complete PubMed/MEDLINE and Embase search strategies and search-yield/de-duplication details.

Additional file 2. PRISMA-ScR checklist. Completed PRISMA-ScR checklist for the scoping review.

## Data Availability

All data supporting the conclusions of this review are derived from published literature cited within the article. The full electronic search strategies are provided in Additional file 1, and the completed PRISMA-ScR checklist is provided in Additional file 2. The anonymised clinical images shown in Figs. 2 and 3 are not publicly available in their original form because of patient privacy considerations, but written informed consent for their publication in the manuscript was obtained.

## Abbreviations

ACL: Anterior cruciate ligament
ATT: Anterior tibial translation
AUC: Area under the curve
GRADE: Grading of Recommendations, Assessment, Development and Evaluations
IKDC: International Knee Documentation Committee
MD-ACL: Mucoid degeneration of the anterior cruciate ligament
MRI: Magnetic resonance imaging
NR: Not reported
NSAIDs: Non-steroidal anti-inflammatory drugs
OAI: Osteoarthritis Initiative
PRISMA-ScR: Preferred Reporting Items for Systematic Reviews and Meta-Analyses extension for Scoping Reviews
PROMs: Patient-reported outcome measures
UKA: Unicompartmental knee arthroplasty
