# Supplementary material for: Mucoid degeneration of the anterior cruciate ligament: a scoping review
Source: BMC Musculoskelet Disord. 2026 Aug 11;27:962. doi: 10.1186/s12891-026-10327-9 (PMC13741440; doi:10.1186/s12891-026-10327-9)
Supplement: Supplementary file 1 [file 12891_2026_10327_MOESM1_ESM.docx]

**Additional file 1**

**Full electronic search strategies**

**Manuscript title:** *Mucoid degeneration of the anterior cruciate ligament: a scoping review*

This file presents the full electronic search strategies executed in PubMed/MEDLINE and Embase for the scoping review on mucoid degeneration of the anterior cruciate ligament (MD-ACL). All searches covered records from database inception to 31 January 2026. No language restriction was applied at the search stage; non-English studies were excluded during eligibility screening as specified in the main manuscript. No publication-type or study-design filters were applied at the search stage in order to maximise sensitivity.

**1. PubMed/MEDLINE**

**Interface:** PubMed (NLM). **Date range:** from database inception to 31 January 2026.

**Search yield:** 117 records.

*Search lines:*

```
#1   "mucoid degeneration"[Title/Abstract] OR "mucinous degeneration"[Title/Abstract] OR "myxoid degeneration"[Title/Abstract] OR "mucoid hypertrophy"[Title/Abstract] OR "mucoid cystic degeneration"[Title/Abstract]

#2   "Anterior Cruciate Ligament"[MeSH Terms] OR "anterior cruciate ligament"[Title/Abstract] OR ACL[Title/Abstract]

#3   #1 AND #2

#4   #3 Filters: from 1900/01/01 to 2026/01/31
```

*Combined query (single-line, copy-ready):*

("mucoid degeneration"[Title/Abstract] OR "mucinous degeneration"[Title/Abstract] OR "myxoid degeneration"[Title/Abstract] OR "mucoid hypertrophy"[Title/Abstract] OR "mucoid cystic degeneration"[Title/Abstract]) AND ("Anterior Cruciate Ligament"[MeSH Terms] OR "anterior cruciate ligament"[Title/Abstract] OR ACL[Title/Abstract])

**2. Embase**

**Interface:** Embase.com (Elsevier). **Date range:** from database inception to 31 January 2026.

**Search yield:** 134 records.

*Search lines:*

```
#1   'mucoid degeneration':ab,ti,kw OR 'mucinous degeneration':ab,ti,kw OR 'myxoid degeneration':ab,ti,kw OR 'mucoid hypertrophy':ab,ti,kw OR 'mucoid cystic degeneration':ab,ti,kw

#2   'anterior cruciate ligament'/exp OR 'anterior cruciate ligament':ab,ti,kw OR acl:ab,ti,kw

#3   #1 AND #2

#4   #3 AND [1900-2026]/py
```

*Combined query (single-line, copy-ready):*

('mucoid degeneration':ab,ti,kw OR 'mucinous degeneration':ab,ti,kw OR 'myxoid degeneration':ab,ti,kw OR 'mucoid hypertrophy':ab,ti,kw OR 'mucoid cystic degeneration':ab,ti,kw) AND ('anterior cruciate ligament'/exp OR 'anterior cruciate ligament':ab,ti,kw OR acl:ab,ti,kw) AND [1900-2026]/py

**Notes:** The /exp operator explodes the Emtree controlled-vocabulary term *'anterior cruciate ligament'* to include all narrower descendants. The :ab,ti,kw field codes restrict free-text matching to abstract, title, and author keywords.

**3. Search yield and de-duplication**

Across the two databases, 251 records were identified (PubMed/MEDLINE n = 117; Embase n = 134). After removal of 99 duplicates, 152 unique records were screened by title and abstract. Eighty-two full-text articles were assessed for eligibility, and 54 publications were included in the final synthesis. The full study-selection flow is shown in Fig. 1 of the main manuscript.

**4. Limits and exclusions applied at search stage**

**Date:** from database inception to 31 January 2026 (inclusive).

**Language:** no restriction at search stage; non-English records were excluded during title/abstract or full-text screening.

**Publication status:** no restriction at search stage; conference abstracts without full-text availability were excluded during screening.

**Study design:** no restriction at search stage; cadaveric and animal-only studies without clinical correlation were excluded during screening.
